# Sulfadiazine Exerts Potential Anticancer Effect in HepG2 and MCF7 Cells by Inhibiting TNFα, IL1b, COX-1, COX-2, 5-LOX Gene Expression: Evidence from In Vitro and Computational Studies

**DOI:** 10.3390/ph17020189

**Published:** 2024-01-31

**Authors:** Mohamed Gomaa, Wael Gad, Dania Hussein, Faheem Hyder Pottoo, Nada Tawfeeq, Mansour Alturki, Dhay Alfahad, Razan Alanazi, Ismail Salama, Mostafa Aziz, Aboelnasr Zahra, Abeer Hanafy

**Affiliations:** 1 Department of Pharmaceutical Chemistry, College of Clinical Pharmacy, Imam Abdulrahman Bin Faisal University, P.O. Box 1982, Dammam 31441, Saudi Arabia; nztawfeeq@iau.edu.sa (N.T.); msalturki@iau.edu.sa (M.A.); 2 Department of Pharmacology, Faculty of Veterinary Medicine, Kafrelsheikh University, Kafr El-Sheikh 6860404, Egypt; wael.gad1@vet.kfs.edu.eg (W.G.); mostafa.mohamed@vet.kfs.edu.eg (M.A.); aboelnasr.zahra@vet.kfs.edu.eg (A.Z.); abeer.ali@vet.kfs.edu.eg (A.H.); 3 Department of Pharmacology, College of Clinical Pharmacy, Imam Abdulrahman Bin Faisal University, P.O. Box 1982, Dammam 31441, Saudi Arabia; fhpottoo@iau.edu.sa; 4 College of Clinical Pharmacy, Imam Abdulrahman Bin Faisal University, P.O. Box 1982, Dammam 31441, Saudi Arabia; 2190004357@iau.edu.sa (D.A.); 2190001320@iau.edu.sa (R.A.); 5 Department of Medicinal Chemistry, Faculty of Pharmacy, Suez Canal University, Ismailia 8366004, Egypt; ismail_mohamed@pharm.suez.edu.eg

**Keywords:** sulfadiazine, anti-inflammatory, anticancer, TNFα, IL1b, COX-1, COX-2, 5-LOX, molecular simulation, drug repurposing, COX/LOX pathway

## Abstract

Drug repurposing is a promising approach that has the potential to revolutionize the drug discovery and development process. By leveraging existing drugs, we can bring new treatments to patients more quickly and affordably. Anti-inflammatory drugs have been shown to target multiple pathways involved in cancer development and progression. This suggests that they may be more effective in treating cancer than drugs that target a single pathway. Cell viability was measured using the MTT assay. The expression of genes related to inflammation (TNFa, IL1b, COX-1, COX-2, and 5-LOX) was measured in HepG2, MCF7, and THLE-2 cells using qPCR. The levels of TNFα, IL1b, COX-1, COX-2, and 5-LOX were also measured in these cells using an ELISA kit. An enzyme binding assay revealed that sulfadiazine expressed weaker inhibitory activity against COX-2 (IC_50_ = 5.27 μM) in comparison with the COX-2 selective reference inhibitor celecoxib (COX-2 IC_50_ = 1.94 μM). However, a more balanced inhibitory effect was revealed for sulfadiazine against the COX/LOX pathway with greater affinity towards 5-LOX (IC_50_ = 19.1 μM) versus COX-1 (IC_50_ = 18.4 μM) as compared to celecoxib (5-LOX IC_50_ = 16.7 μM, and COX-1 IC_50_ = 5.9 μM). MTT assays revealed the IC_50_ values of 245.69 ± 4.1 µM and 215.68 ± 3.8 µM on HepG2 and MCF7 cell lines, respectively, compared to the standard drug cisplatin (66.92 ± 1.8 µM and 46.83 ± 1.3 µM, respectively). The anti-inflammatory effect of sulfadiazine was also depicted through its effect on the levels of inflammatory markers and inflammation-related genes (TNFα, IL1b, COX-1, COX-2, 5-LOX). Molecular simulation studies revealed key binding interactions that explain the difference in the activity profiles of sulfadiazine compared to celecoxib. The results suggest that sulfadiazine exhibited balanced inhibitory activity against the 5-LOX/COX-1 enzymes compared to the selective COX-2 inhibitor, celecoxib. These findings highlight the potential of sulfadiazine as a potential anticancer agent through balanced inhibitory activity against the COX/LOX pathway and reduction in the expression of inflammatory genes.

## 1. Introduction

Cancer stands as a primary contributor to global mortality, causing millions of deaths annually [1]. The World Health Organization (WHO) projects a significant surge in cancer incidence in the coming decades, with expectations of over 20 million new cases per year by 2025 [2]. Managing this disease remains a challenge, partially due to the growing resistance to medications and the adverse health impacts of chemotherapy, both during and after treatment [3]. Current anticancer drugs often come with substantial side effects, and the quest for safer and more effective alternatives is neither swift nor cost effective. The process of discovering novel drugs is a laborious endeavor, typically taking approximately 10–15 years and costing tens of millions of dollars on average.

To address the increasing global disease burden, it is crucial to develop alternative strategies for discovering new therapeutics that are efficient in terms of time and cost. One highly appealing approach to achieve this goal is the concept of drug repurposing. Drug repurposing, also known as drug repositioning or therapeutic switching, involves identifying new therapeutic uses for existing approved drugs, regardless of their originally intended purpose [4]. The advantage of drug repurposing lies in the fact that the candidate drugs already have well-documented pharmacokinetics, pharmacodynamics, and toxicity profiles. This characteristic streamlines the discovery process, making it more time and cost effective and reducing the associated risks compared to traditional drug discovery methods [4]. Remarkably, drug repurposing boasts a high success rate, with an estimated 30% of FDA-approved drugs and vaccines being repurposed drugs [4]. Therefore, there is a compelling need to further develop platforms and engage in research utilizing in silico methodologies, translational studies, and clinical investigations to expedite the drug discovery process.

Notable examples of successful drug repurposing include sildenafil (commonly known as Viagra, developed by Pfizer), which was originally designed as an antiangina medication but was later approved for the treatment of erectile dysfunction. Another example is atomoxetine, initially developed as an antidepressant but subsequently approved for managing attention deficit hyperactivity disorder (ADHD) [5]. Additionally, thioridazine, an antipsychotic medication originally used for schizophrenia, has shown promising selectivity in targeting cancer stem cells and impacting survival rates in chronic lymphocytic leukemia [6].

A recent and compelling illustration of drug repurposing is in the context of the coronavirus disease 2019 (COVID-19) pandemic. Rigorous efforts to repurpose antiviral drugs for COVID-19 have been successful, leading to the recommendation of antivirals like remdesivir and ribavirin for the therapeutic management of moderate to severe SARS-CoV-2 infections [7].

Cyclooxygenases (COX) and lipoxygenase (LOX), particularly the COX-2 and 5-LOX pathways, represent highly compelling and relevant targets for cancer therapy [8,9,10,11]. COX enzymes play a pivotal role as rate-limiting enzymes, converting arachidonic acids into prostaglandins (PGs), which are crucial for the regulation of various bodily functions, including the gastric mucosa, platelet function, and renal blood flow. There are two distinct isozymes of COX: COX-1 and COX-2. COX-1 is expressed in a wide range of normal tissues and is primarily responsible for prostaglandin production. In contrast, COX-2 tends to be overexpressed in inflammatory and neoplastic tissues and has been implicated in both inflammatory processes and the development of cancer [12,13]. COX-2 has been linked to multiple stages of cancer progression and is notably highly expressed in certain human cancers, especially in cases of colon cancer [8,9,10]. Utilizing non-steroidal anti-inflammatory drugs (NSAIDs) and inhibiting COX-2 activity have been associated with antitumor and antiangiogenic effects in various human cancers [8,9,10]. Furthermore, NSAIDs have been reported to reduce the risk of developing several common solid tumors, including breast, colon, lung, and prostate cancer [14,15].

Presently, current COX-based therapeutics, encompassing both selective and non-selective COX inhibitors, are primarily employed for managing inflammatory conditions. These two classes of drugs exhibit similar efficacy but differ in their profiles of side effects [12]. Non-selective COX inhibitors, such as diclofenac, are linked to a higher incidence of side effects concerning the heart, kidney, and renal function, and are most commonly associated with an elevated risk of gastrointestinal ulceration and bleeding [11,12]. In contrast, selective COX-2 inhibitors like celecoxib provide comparable analgesic, antipyretic, and anti-inflammatory effects while being associated with fewer gastrointestinal, renal, and cardiovascular side effects [12].

Of note, a recent study explored the use of celecoxib, a selective COX-2 inhibitor, as part of a repurposing strategy to address therapeutic resistance in BRAFV600E colorectal cancer [16]. While the utilization of COX-2 inhibitors as potential anticancer agents shows promise, the presence of cardiovascular and gastrointestinal side effects associated with COX inhibition may pose significant challenges to this approach.

5-LOX is a crucial enzyme responsible for generating pro-inflammatory compounds known as leukotrienes (LTs), constituting the second primary category of derivatives from arachidonic acid, and it plays a significant role in the inflammatory cascade [11]. Various leukotrienes produced by the LOX pathway, such as LTB4, have been demonstrated to promote tumorigenesis and are linked to conditions like lung, esophageal, and prostate cancer. Consequently, targeting this pathway holds promise for halting the progression of cancer [17]. The simultaneous inhibition of both the COX and 5-LOX pathways is anticipated to yield a more potent anti-inflammatory effect, expanding the range of potential anticancer activities. This strategy presents a highly encouraging avenue for the development of innovative and comparatively safer chemotherapeutic agents for cancer [18,19,20].

Sulfonamides represent a significant class of synthetic antimicrobial medications known for their utility as broad-spectrum antibacterial agents. They function as competitive antagonists and structural analogs of *p*-aminobenzoic acid (PABA). Their primary mode of action involves the inhibition of folic acid synthesis, a critical process for bacterial DNA replication [21]. Sulfonamides find extensive use in the treatment of various infections, including urinary tract and upper respiratory infections, and they have garnered approval for a wide array of additional medical indications owing to their safety, efficacy, and favorable pharmacological characteristics [21].

In a study conducted by Wan et al., a range of sulfonamide derivatives exhibited anticancer properties and subsequently received FDA approval for cancer treatment [22]. Notably, belinostat, an inhibitor of histone deacetylase (HDAC), was sanctioned for the treatment of T-cell lymphoma, while ABT-199, a highly selective inhibitor of Bcl-2, was approved for patients with chronic lymphocytic leukemia (CLL) exhibiting a 17p chromosomal deletion [22]. Another intriguing example is indisulam, which is presently undergoing clinical trials for the treatment of high-risk neuroblastoma, with its anticancer potential initially uncovered through a drug repurposing approach [23].

Additionally, a sulfonamide derivative, sulfasalazine, serves as a disease-modifying antirheumatic drug (DMARD) utilized in managing autoimmune conditions like rheumatoid arthritis and inflammatory bowel disease. It also has off-label applications in the treatment of individuals with conditions such as ankylosing spondylitis, Crohn’s disease, psoriasis, and psoriatic arthritis [24]. Sulfasalazine functions as an azo prodrug, undergoing reduction by bacterial azoreductases in the lower intestine to produce active metabolites, sulfapyridine and mesalazine (5-aminosalicylic acid) [24,25].

**Figure 1:**
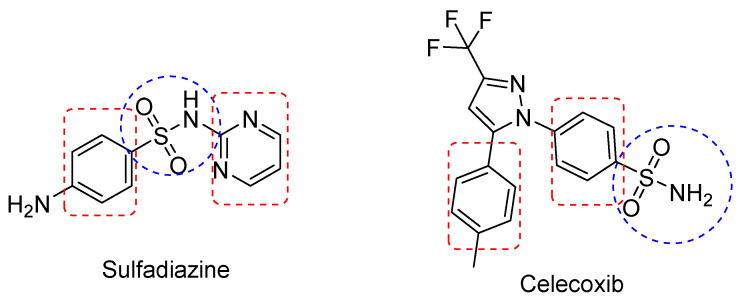
The chemical structure of sulfadiazine and celecoxib showing common structural features.

Furthermore, a structural analysis revealed that antibacterial sulfonamides, including sulfadiazine, exhibit certain structural similarities with celecoxib, the standard COX-2 inhibitor (Figure 1). These included the sulfonamide group and the cis biphenyl structure.

Given the emerging insights into the involvement of the COX/LOX axis in cancer pathogenesis and the success of several sulfonamide derivatives as anticancer therapeutics, our research aims to delve deeper into the potential anticancer properties of the sulfonamide antibiotic sulfadiazine. We seek to uncover the mechanism through which this antibacterial agent operates, particularly its capacity to inhibit the COX/LOX pathway. Our goal is to assess its repurposing suitability as a potential anticancer effect.

## 2. Results and Discussion

### 2.1. Effects of Sulfadiazine on In Vitro COX-1, COX-2 and 5-LOX Activities

In vitro inhibition studies were conducted to assess the capability of sulfadiazine to inhibit ovine COX-1, human recombinant COX-2, and rabbit 5-LOX. The data presented in Table 1 indicate that sulfadiazine exhibited relatively weaker inhibitory activity against the COX-1 isozyme (IC_50_ = 18.4 μM) and had comparable inhibitory activity against the COX-2 isozyme (IC_50_ = 5.27 μM) and against the 5-LOX isozyme (IC_50_ = 19.1 μM) when compared to the COX-2 selective reference drug celecoxib (COX-1 IC_50_ = 5.9 μM, COX-2 IC_50_ = 1.94 μM, and 5-LOX IC_50_ = 16.7 μM) (Table 1). 

### 2.2. Effects of Sulfadiazine on Cell Viability of HepG2, MCF7, and THLE2 Cells

The cytotoxicity of sulfadiazine was assessed through a cell-based assay involving HepG2 (human liver cancer), MCF7 (human breast cancer), and THLE2 (healthy human liver) cells. The IC_50_ of sulfadiazine in different cell types was determined using the MTT assay. The cytotoxic impact of sulfadiazine on three cell types, human liver cancer HepG2, human breast cancer MCF7, and healthy human liver THLE2 cells, was determined by exposing these cells to different concentrations of sulfadiazine (100, 50, 25, 12.5, and 3.25 μg/mL) for a duration of 24 h, and cell viability was subsequently determined using the MTT assay (Table 2).

The MTT assay results revealed a significant dose-dependent antiproliferative effect of sulfadiazine on HepG2 and MCF7 cells, with IC_50_ values of 245.69 ± 4.1 µm, 215.68 ± 3.8 µm, respectively. These values were compared to vehicle (DMSO)-treated MCF7 cells (as shown in Figure 1). However, it is important to note that the cytotoxic effects of sulfadiazine were less potent than those of the standard anticancer agent cisplatin, which exhibited lower IC_50_ values in HepG2 and MCF7 cell lines (66.92 ± 1.8 µm and 46.83 ± 1.3 µm, respectively).

Interestingly, no cytotoxic effects were observed for sulfadiazine on normal liver cells (IC_50_ = 4159 ± 90.5 µm), while cisplatin exhibited slightly higher cytotoxicity in normal liver cells (IC_50_ = 2144 ± 95.3 µm) in comparison to sulfadiazine. These results suggest an antiproliferative effect of sulfadiazine on HepG2 and MCF7 cells, possibly through the inhibition of the COX-2/PGE2 signaling axis. Although this anticancer effect is less potent than that of cisplatin, it appears that sulfadiazine is safer for normal cells.

Furthermore, the results indicated that sulfadiazine was not cytotoxic at concentrations of up to 1/10th of its IC_50_.

However, cell viability showed a slight decrease at higher concentrations. As a result, for all subsequent experiments, 1/10 of the IC_50_ of sulfadiazine was selected for treatment.

### 2.3. Effect of Sulfadiazine on the Levels of Inflammation-Related Parameters in LPS-Inflamed Cells

Lipopolysaccharide (LPS) treatment triggers a sequence of intracellular signaling events in both cancerous and normal cells, culminating in the production of cytokines and other inflammatory mediators that initiate a proinflammatory response [26]. Using HepG2, MCF7, and THLE2 cells provides a suitable model for assessing the potential of a drug candidate to exhibit anti-inflammatory properties [27]. LPS is known to induce an inflammatory cascade through the activation of MAPKs [27,28]. It has been observed that mice lacking MAPKs tend to produce higher levels of TNFα, IL-6, and IL-12. Additionally, the expression of COX-2 has been found to be sensitive to p38 MAPK blockade, and its regulation may depend on MAPK activation of the NF-κB pathways, underscoring their potential as therapeutic targets for treating inflammatory diseases.

**Figure 2:**
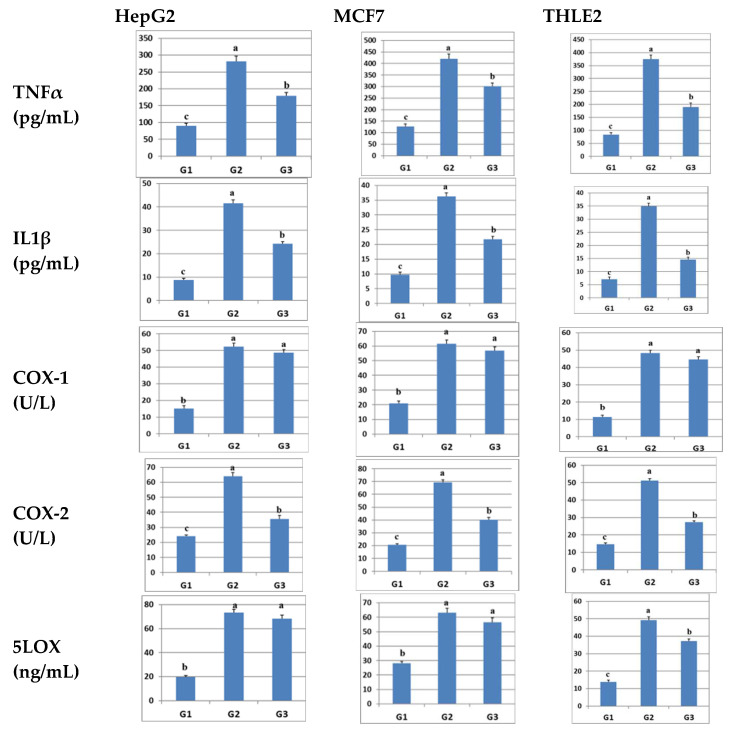
Illustration of the impact of sulfadiazine on the levels of inflammation-related parameters, including TNFα, IL1b, COX-1, COX-2, and 5LOX, in LPS-inflamed HepG2, MCF7, and THLE2 cells. The data are presented as mean ± SE (n = 5). Different superscript letters (a–c) within columns indicate significant differences (*p* ≤ 0.05). The groups are labeled as follows: G1 (control, vehicle-treated cells), G2 (untreated LPS-inflamed cells), and G3 (sulfadiazine-treated LPS-inflamed cells).

In the current study, sulfadiazine was tested for its ability to inhibit pro-inflammatory mediators, including TNFα, IL1b, COX-1, COX-2, and 5-LOX in LPS-inflamed HepG2, MCF7, and THLE-2 cells. The data presented in Figure 2 indicate a notable increase in the levels of all these pro-inflammatory mediators in untreated LPS-inflamed cells (G2) when compared to control (vehicle-treated) cells (G1). However, these elevated levels were significantly reduced following treatment with sulfadiazine (G3), with the exception of COX-1 levels in all cells and 5-LOX in HepG2 and MCF7 cells. Significant differences were observed between the various groups, and the reduced levels of pro-inflammatory mediators in G3 remained significantly higher than those in G1. These results signify that sulfadiazine possesses the potential to inhibit inflammation induced by LPS in different cell types, specifically in both cancer cells (HepG2, MCF7) and normal cells (THLE2). This inhibition is primarily achieved by suppressing the release of pro-inflammatory mediators, notably TNFα, IL1b, and COX-2 enzyme expression (Figure 2).

### 2.4. Effect of Sulfadiazine on the Expression of Inflammation-Related Genes in LPS-Inflamed Cells

The results mentioned above demonstrate that sulfadiazine has the capacity to suppress the inflammatory response at the molecular level, affecting key inflammatory mediators such as TNFα and IL1b, as well as the levels of enzymes COX-1, COX-2, and 5-LOX.

**Figure 3:**
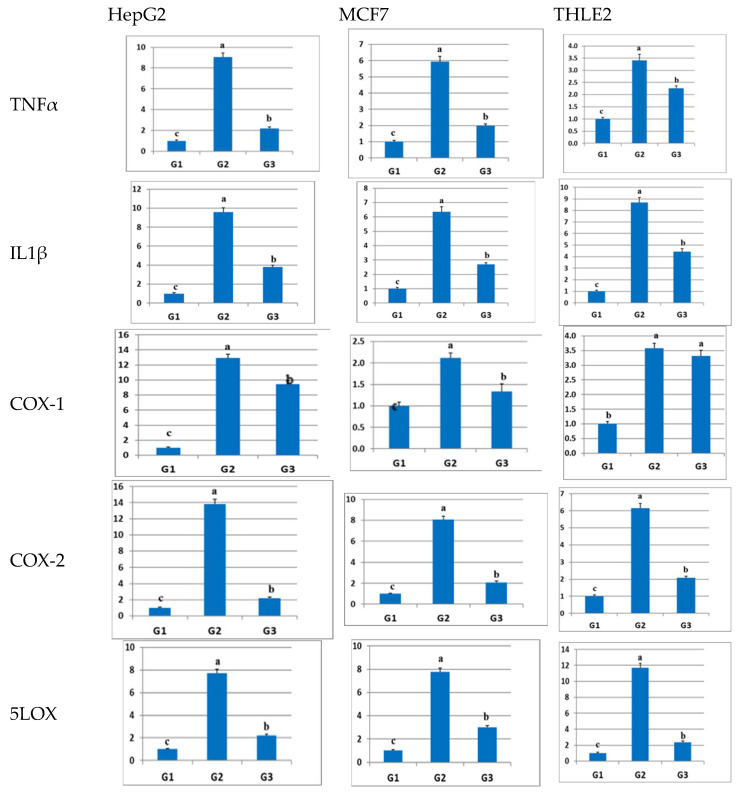
The impact of sulfadiazine on the expression of inflammation-related genes, including TNFa, IL1b, COX-1, COX-2, and 5LOX, in LPS-inflamed HepG2, MCF7, and THLE2 cells. The housekeeping gene encoding β-actin served as an internal reference, and the data are presented as mean ± SE (n = 5). Different superscript letters (a–c) within columns indicate significant differences (*p* ≤ 0.05). The expression level of the target genes is represented in this figure.

To further validate this effect on a molecular level, real-time PCR (qPCR) was employed to assess the relative gene expression of inflammation-related genes, including TNFα, IL1b, COX-1, COX-2, and 5-LOX in HepG2, MCF7, and THLE-2 cells. The transcription levels of these genes were examined after treatment with sulfadiazine. The qPCR results obtained (as shown in Figure 3) indicated a significant (*p* ≤ 0.05) upregulation in the expression levels of all these inflammation-related genes in all LPS-inflamed cells (G2) in comparison to the control group (G1), as illustrated in Figure 3 (further data and linear amplification curves are available in the Supplementary Materials). The increased expression of these inflammation-related genes was significantly blunted after the administration of sulfadiazine (G3) in all cell types, with the exception of COX-1 in THLE2 cells, where the difference was not significant compared to G2. However, in THLE2 cells, the expression levels in the sulfadiazine-treated group remained significantly higher than those in the control (untreated) group (G1). In conclusion, these results confirm the anti-inflammatory effect of sulfadiazine at both the gene and protein levels.

### 2.5. Effect of Sulfadiazine on the Levels of MDA and Antioxidant Enzymes in LPS-Inflamed Cells

The interplay between inflammatory pathways and oxidative stress is widely recognized as a key factor in the development of numerous medical conditions. Many natural and synthetic compounds with anti-inflammatory properties also exhibit antioxidative characteristics. To investigate whether sulfadiazine possesses antioxidant properties, we conducted an examination of the alterations in the lipid peroxidation marker MDA, which serves as an indicator of oxidative stress, as well as the activities of antioxidant enzymes, specifically SOD, CAT, and GPX, in HepG2, MCF7, and THLE-2 cells after exposure to sulfadiazine.

**Figure 4:**
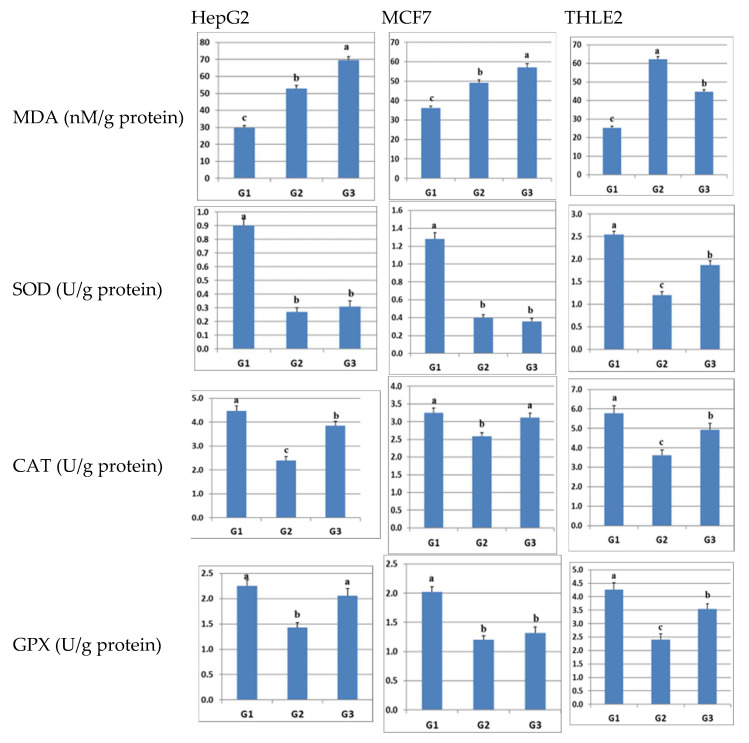
Illustration of the impact of sulfadiazine on the oxidative stress (MDA) and antioxidant (SOD, CAT, GPX) status in HepG2, MCF7, and THLE2 cells subjected to LPS-induced inflammation. The data are presented as the mean ± standard error (n = 5). Different superscript letters (a–c) within columns indicate statistically significant differences (*p* ≤ 0.05). The experimental groups are denoted as follows: G1 (control, vehicle-treated cells), G2 (untreated LPS-inflamed cells), and G3 (sulfadiazine-treated LPS-inflamed cells).

The outcomes depicted in Figure 4 disclosed a statistically significant increase (*p* ≤ 0.05) in MDA levels and a notable decrease in the levels of SOD, CAT, and GPX in all cells subjected to LPS-induced inflammation (group G2) when compared to the control group (group G1). In the case of cancer cells (HepG2 and MCF7), the administration of sulfadiazine (group G3) led to a significant augmentation in MDA levels, as well as increased levels of SOD, CAT, and GPX, with the exception of SOD in the two cancer cell lines and GPX in MCF7 cells, relative to the levels observed in group G2. Conversely, within normal liver cells (THLE2), the introduction of sulfadiazine resulted in a substantial reduction in MDA levels and a significant elevation in the levels of SOD, CAT, and GPX when compared to group G2.

Collectively, these findings suggest that sulfadiazine exerts an antioxidative influence on LPS-inflamed HepG2, MCF7, and THLE2 cells. Nevertheless, it is crucial to recognize that the effects of sulfadiazine may diverge based on the specific type of cells involved. For instance, in cancer cells, sulfadiazine appears to induce oxidative stress, as indicated by the increase in MDA levels, potentially leading to the initiation of apoptosis in cancer cells. In contrast, in normal cells (THLE2), sulfadiazine appears to reduce MDA levels while bolstering the activity of antioxidant enzymes, implying a capacity for scavenging free radicals in these cells.

### 2.6. Computational Studies

The initial investigation involved examining the COX/LOX inhibition profiles of eight antibacterial sulfonamides, including sulfadiazine. This analysis began with docking these sulfonamides into the active sites of the specified targets. Their binding patterns, interactions with the targets, and binding strengths were then scrutinized and compared to those of the reference COX-2 selective drug, celecoxib.

To ensure the accuracy of the docking procedure, it was validated by redocking the ligands that were co-crystallized with their respective targets (COX-1 and COX-2). The same docking procedure and protocol used for screening the potential sulfonamide ligands were applied. Subsequently, the predicted lowest energy conformations for each target were superimposed with their corresponding crystallized ligands using Maestro’s structure superposition tool. The classical root mean square deviation (RMSD) for the predicted binding poses was calculated. An RMSD value of less than 2 Å is widely accepted as an effective threshold for validating the correct positioning of molecules.

The results indicated a good superimposition of binding modes with RMSD values of 1.8524 Å for COX-1 and 1.3266 Å for COX-2, which initially confirmed the accuracy of Glide in predicting the binding poses for the COX inhibitors. Subsequently, the antibacterial sulfonamide ligands were docked in the active sites of COX-1 (PDB: WBE), COX-2 (PDB: 3LN1), and 5-LOX (PDB: 3V99) by using the extra precision mode in Glide.

**Figure 5:**
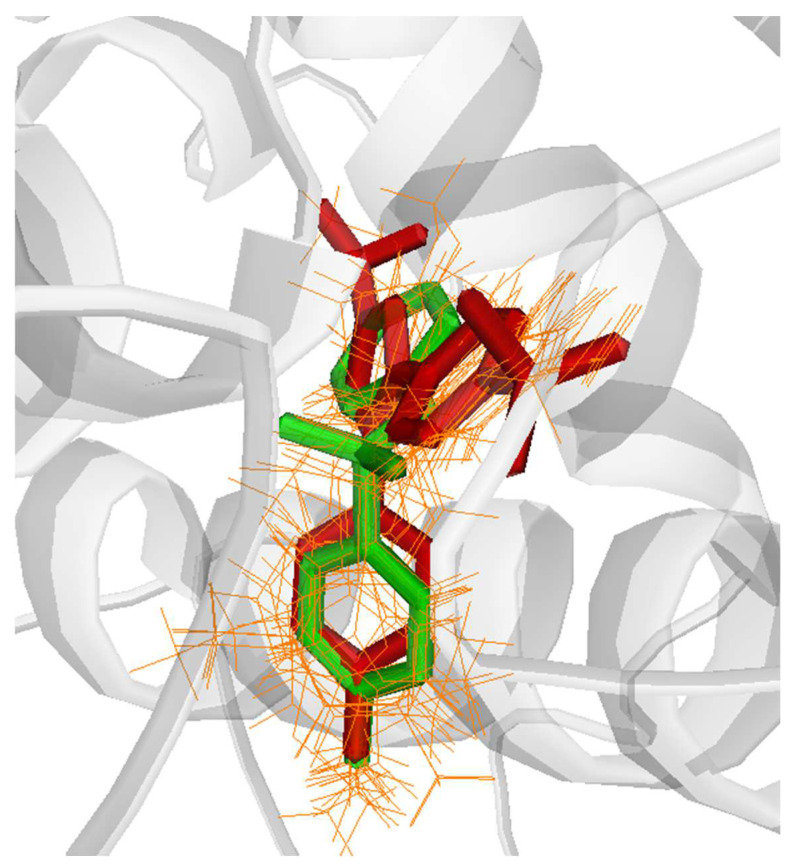
Comparative binding positions of sulfadiazine (green) celecoxib (red) and selected antibacterial sulfonamides (orange) bound to COX-2 active site (PDB: 3LN1).

The findings demonstrated that all sulfonamides exhibited a relatively similar docking pattern in the COX-2 active site, closely resembling the binding pattern of the reference drug, celecoxib (Figure 5). This suggests a potential affinity of these compounds for the COX-2 active site. The calculated RMSD values for the best binding poses, in comparison to sulfadiazine, ranged from 1 to 5 Å for sulfamerazine and sulfabenzamide, respectively.

The analysis involved evaluating the relative binding affinities of the docked compounds with respect to the three enzymes. The findings indicated that sulfadiazine achieved the highest Glide score for inhibiting 5-LOX and the second highest score for inhibiting COX-2, following benzamide. Conversely, it secured the second lowest score for COX-1 inhibition, coming just above sulfaguanidine in ranking. This particular inhibition profile is noteworthy due to its emphasis on COX-2 and 5-LOX inhibition. Such a profile suggests the potential for a drug candidate that may be better tolerated in clinical settings, and offers the possibility of broader applications and indications (as detailed in Table 3).

The assessment of the computed binding affinities for all sulfonamides on the three target enzymes revealed a trend in which inhibition was generally more evenly distributed and leaned towards 5-LOX, at the expense of COX-1, when compared to the reference compound, celecoxib. Specifically, sulfadiazine displayed the highest selectivity for COX-2 over COX-1 (with a ratio of 1.25) and for 5-LOX over COX-1 (with a ratio of 1.08), as opposed to the respective ratios of 1.16 and 0.53 for COX-2 over COX-1 and 5-LOX over COX-1, respectively.

Biological testing demonstrated the anti-inflammatory and antiproliferative effects of sulfadiazine. The competitive enzyme binding assay showed that sulfadiazine has a more balanced inhibitory activity on COX-1/COX-2/5-LOX than the reference drug celecoxib.

Table 4 shows that celecoxib has better affinity to COX-2 than sulfadiazine with an IC_50_= 1.94 μM compared to IC_50_= 5.27 μM for sulfadiazine. The experimental and calculated binding affinities for COX-2 were in close agreement, with celecoxib being nearly two times more active than sulfadiazine and presenting almost the same selectivity over COX-1 but higher selectivity over 5-LOX. This was consistent in both the experimental and calculated values. The experimental COX-2/COX-1 selectivity for sulfadiazine was found to be 3.6 compared to 2.9 for celecoxib, and the calculated COX-2/COX-1 selectivity was 1.3 and 1.2 for sulfadiazine and celecoxib, respectively. On the other hand, the experimental 5-LOX/COX-1 selectivity was 1 and 0.3 for sulfadiazine and celecoxib, respectively, and the calculated selectivity was 1.1 for sulfadiazine and 0.5 for celecoxib. This indicates that sulfadiazine exhibited higher COX-2/COX-1 selectivity without an increase in COX-2 affinity when compared to celecoxib, while the activity profile favored 5-LOX inhibition at the expense of COX-1 inhibition. These results are comparable to the celecoxib derivative incorporating a N-difluoromethyl-1,2-dihydropyridine-2-one LOX pharmacophore, which exhibited dual COX and 5-LOX inhibitory capacity (COX-2 IC_50_ = 0.69 μM, COX-1 IC_50_ = 13.1 μM; 5-LOX IC_50_ = 5.0 μM) [29].

In terms of binding interactions, sulfadiazine showed specific binding types that potentially made it a more balanced inhibitor for the three targets. In the COX-2 active site, sulfadiazine did not appear to mainly interact via its sulfonamide with the selectivity region-1 formed by His 75 and Arg 499, which is similar to celecoxib.

**Figure 6:**
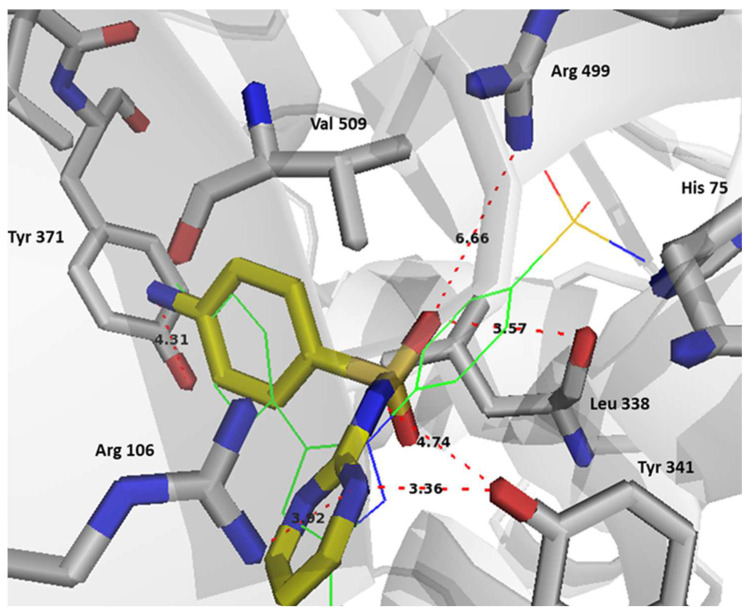
Binding interactions of sulfadiazine (stick representation, carbon in yellow) and celecoxib (line representation, carbon in green) with COX-2 active site (PDB: 3LN1). Distances are represented as red dotted lines and are measured in Angstrom.

However, this was compensated through the selective binding of its sulfonamide and pyrimidine nitrogens with Tyr 341 and Arg 106, as well as through potential binding with Arg 499 through a coordinating water molecule (Figure 6). The primary amino group of sulfadiazine, like the methyl group of celecoxib, occupied the selectivity region-2 and utilized the active site volume created by the orientation of Leu 370 side chain that points out of the active site in COX-2, while it points into the active site in COX-1. A better binding interaction is sought with sulfadiazine in this region over celecoxib through potential binding with Tyr 371. The overall binding pattern and interaction profile suggest that celecoxib has better affinity to COX-2 over sulfadiazine, which was also reflected in their Glide scores (Table 4).

**Figure 7:**
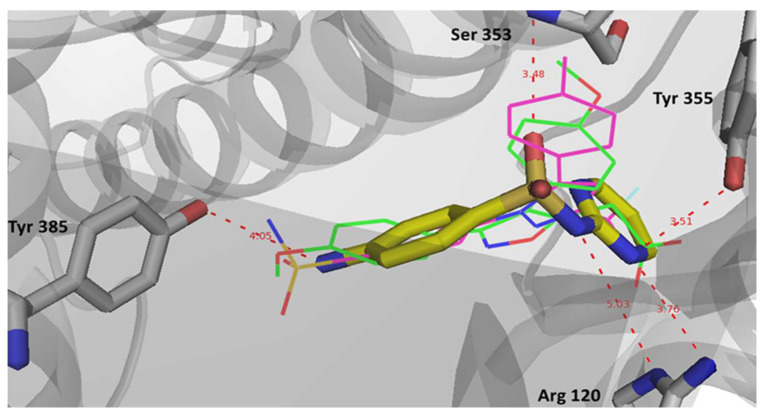
Binding interactions of sulfadiazine (stick representation, carbon in yellow), celecoxib (line representation, carbon in magenta), and mofezolac (line representation, carbon in green) with COX-1 active site (PDB: 5WBE). Distances are represented as red dotted lines and are measured in Angstrom.

In COX-1, sulfadiazine showed a docking pose comparable to that of celecoxib and the crystallized inhibitor, mofezolac. Sulfadiazine was able to bind the key residue Arg 120 specifically with its sulfonamide and pyrimidine nitrogens again, while celecoxib binds with Arg120 at a lower affinity via its pyrazole ring (Figure 7). The potent non-selective COX inhibitor mofezolac binds this key residue with its carboxylate group through strong electrostatic interactions. This finding supports the higher selectivity of celecoxib and sulfadiazine to COX-2. Potential electrostatic interactions were also noted with Ser 353, Tyr 355. There is also potential binding with Tyr 385, where celecoxib and mofezolac appear to bind with their sulfonamide and methoxy groups, respectively.

**Figure 8:**
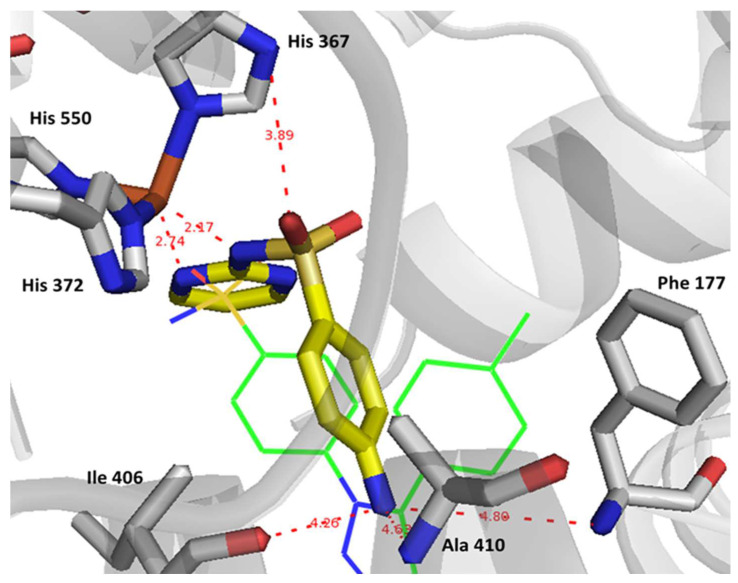
Binding interactions within the 5-LOX active site (PDB: 3V99) involving sulfadiazine (depicted in stick representation with carbon atoms in yellow) and celecoxib (depicted in line representation with carbon atoms in green). Distances between interacting components are indicated with red dotted lines and are quantified in Angstrom units.

Remarkably, in the case of 5-LOX, sulfadiazine demonstrated a notable capability to coordinate with the Fe^+3^ ion present in the enzyme’s active site. This coordination process plays a pivotal role in initiating the oxidation of the natural substrate, arachidonic acid, a crucial step in the biosynthesis of prostaglandins. Sulfadiazine achieves this coordination through the participation of its sulfonamide and pyrimidine nitrogens, as illustrated in Figure 8. In contrast, celecoxib exhibited a weaker interaction with Fe^+3^ due to its less basic sulfonamide oxygen and a longer bond length. Additionally, the presence of an aniline moiety in sulfadiazine facilitated a more secure anchoring of the drug within the active site, potentially forming bonds with Phe 177 and its primary amine with Ile 406 and Ala 410. This explains why sulfadiazine displayed a higher affinity for 5-LOX when compared to celecoxib, ultimately leading to a more balanced effect on all three target enzymes.

To assess the relative binding stability and affinity of sulfadiazine, molecular dynamics simulations were conducted using its best poses within the binding pockets of the three enzymes. Despite limitations, such as the requirement for lengthy simulation periods and huge computational capacity and specifications, in order to attain accurate results about a given systems dynamic properties and binding characteristics, MD simulations have been shown to accurately predict dynamic profiles that are comparable with experimental results. These are critical for the subsequent substantiation of predicted binding modes, properties, and affinities, and are highly useful in drug discovery [30,31,32]. 

Typically, the molecular deviation of a specific ligand concerning a specified reference structure is assessed using the RMSD (Root Mean Square Deviation) as an analytical parameter. This metric serves as a dependable tool for confirming both the stability of the ligand within the target and the reliability of the applied molecular dynamics (MD) simulation protocol. A high RMSD value suggests instability in the target and significant conformational changes [33]. Furthermore, high RMSD values are indicative of limited ligand–target affinity, signifying that the ligand is unable to remain bound within the canonical binding site of the target throughout the duration of the simulation [34].

**Figure 9:**
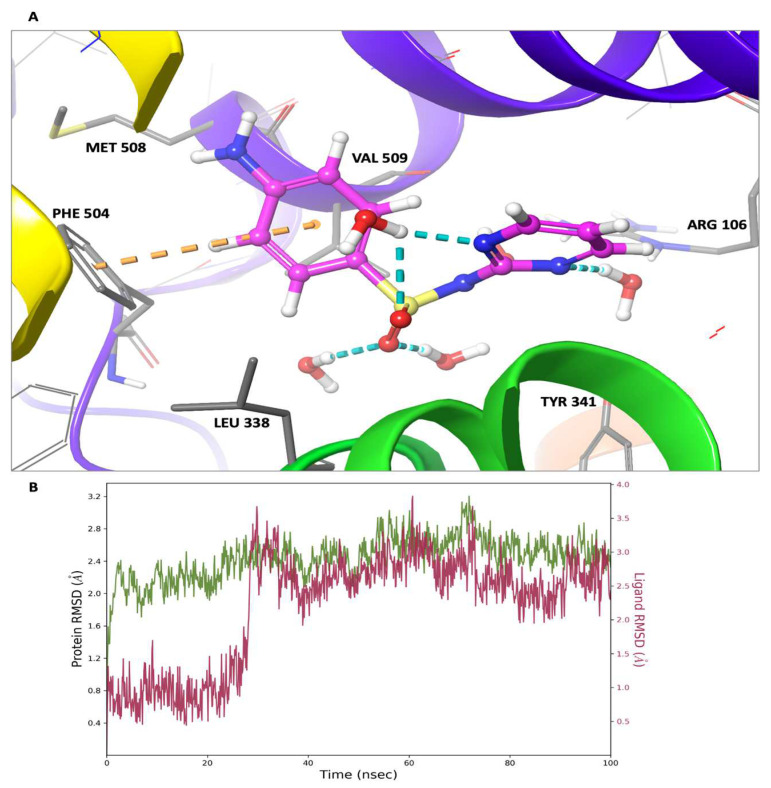
Molecular dynamics (MD) simulation findings: sulfadiazine’s interaction profile in the COX-2 binding pocket. (**A**) Depiction of the final MD simulation frame, showcasing sulfadiazine within the COX-2 binding pocket. All bonds are represented with dotted lines: hydrogen bonds are denoted in blue, aromatic hydrogen bonds in green, and pi–pi stacking interactions in orange. (**B**) Graph displaying the Root Mean Square Deviation (RMSD) for sulfadiazine within the COX-2 binding pocket. The green line illustrates fluctuations in the protein backbone relative to the initial reference point, while the red line portrays the ligand’s fluctuations.

**Figure 10:**
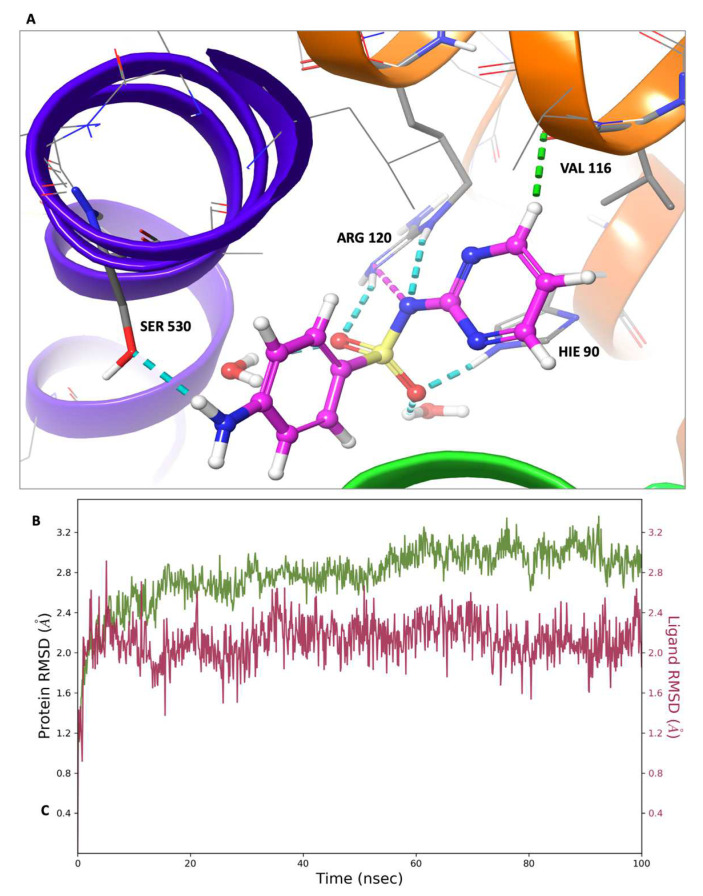
Molecular dynamics (MD) simulation results: sulfadiazine’s interaction profile within the COX-1 binding pocket. (**A**) Depiction of the final MD simulation frame, illustrating sulfadiazine within the COX-1 binding pocket. All bonds are represented with dotted lines, with hydrogen bonds in blue and aromatic hydrogen bonds in green. The binding residues are labeled, and the iron (Fe^+2^) ion is depicted as a rust-colored sphere. (**B**) Graph depicting the Root Mean Square Deviation (RMSD) for sulfadiazine in the 5-LOX binding pocket. The green line represents fluctuations in the protein backbone relative to the initial reference point, while the red line represents the ligand’s fluctuations.

**Figure 11:**
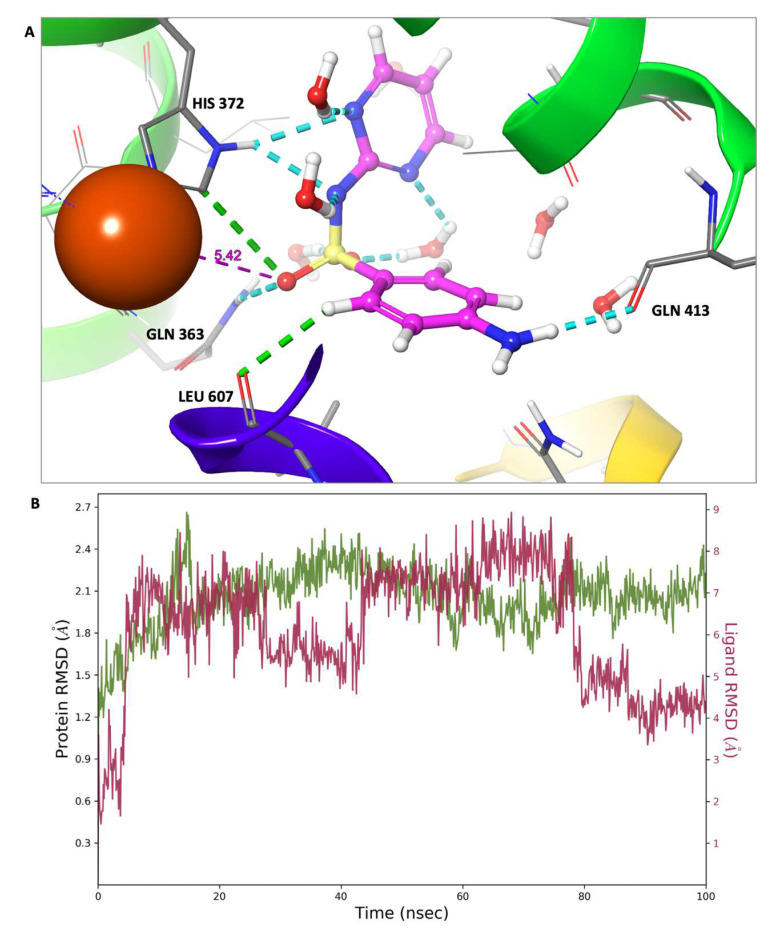
Molecular dynamics (MD) simulation results: sulfadiazine’s interaction profile within the 5-LOX binding pocket. (**A**) Visualization of the final MD simulation frame, featuring sulfadiazine situated within the 5-LOX binding pocket. Dotted lines represent all bonds, with hydrogen bonds in blue and aromatic hydrogen bonds in green. The binding residues are labeled, and the iron (Fe^+2^) ion is illustrated as a rust-colored sphere. (**B**) Graph depicting the Root Mean Square Deviation (RMSD) for sulfadiazine within the 5-LOX binding pocket. The green line represents fluctuations in the protein backbone relative to the initial reference point, while the red line reflects the ligand’s fluctuations.

In this investigation, a molecular dynamics simulation with a cumulative duration of 100 nanoseconds was conducted on the solvated complexes formed by sulfadiazine with COX-2, COX-1, and 5-LOX. The primary objective was to assess the endurance and stability of the binding conformations throughout the simulation period. The RMSDs of Cα (root mean square deviations of the alpha carbon atoms) for the solvated receptors in conjunction with sulfadiazine remained constant and exhibited stability throughout the entire simulation duration, as depicted in Figure 9, Figure 10 and Figure 11.

Moreover, in each of the complexes, sulfadiazine maintained a high degree of stability, exhibiting an average deviation of only 2–4 Å from the initial reference point throughout the entire simulation duration. This consistent behavior revealed no significant alterations in the initial bond configuration. The observed resilience of sulfadiazine within the central pocket of COX-2, COX-1, and 5-LOX can be attributed to the presence of the 2-sulfamoylamino pyrimidine group, which engages in hydrogen bonding, aromatic hydrogen bonding, and pi–pi stacking interactions with crucial amino acid residues. An RMSD analysis of the core domain of COX-1 and 5-LOX indicated that certain pivotal amino acid residues exhibited minor and well-regulated fluctuations while shaping the binding pocket for sulfadiazine.

In contrast to molecular docking, which took place in a vacuum, molecular dynamics simulations were conducted in an aqueous environment. Notably, the iron ion within 5-LOX played a significant role in stabilizing the complex by coordinating with two water molecules and moving closer to and binding with His 372. Initially, His 372 was responsible for binding the iron, forming two hydrogen bonds in the process, making the iron available for further binding. The sulfonamide oxygen also drew nearer to the iron and had the potential to participate in iron binding. This interaction may partially account for sulfadiazine’s selectivity towards 5-LOX.

Additionally, the contributing residues within 5-LOX are predominantly hydrophobic in nature. This observation aligns with a recent finding in the current literature, which characterizes the 5-LOX pocket as primarily hydrophobic, characterized by its depth and a conserved U-shaped hydrophobic configuration [35]. Taken together, this supports the robust stability observed in the interaction profile, as evidenced through the RMSD analysis.

Molecular simulation studies conclusively show that there are substantial key binding interactions capable of describing the difference in the activity profiles of sulfadiazine compared to celecoxib. Sulfadiazine appears to be a more selective COX-2 inhibitor but with more inhibitory activity on 5-LOX than COX-1. The key binding group in sulfadiazine was found to be the 2-sulfamoylamino pyrimidine group, which was involved in binding in all three targets. 

## 3. Materials and Methods

### 3.1. Apparatus, Cell Lines and Chemicals

An ELISA reader (StatFax-2100, Awareness Technology, Inc., Palm City, FL, USA) and CO_2_ incubator (SHEL LAB, Sheldon Manufacturing NC., Cornelius, OR, USA) were used. The Q5000 (Uv-Vis spectrophotometer Q5000, Quawell, San Jose, CA, USA) was used for quantification of the concentration of RNA and cDNA, and StepOnePlus real-time thermal cycler (Applied Biosystems, Life technology, Carlsbad, CA, USA) was used for qPCR. Human hepatoma HepG2, breast cancer MCF7, and normal liver THLE2 cell lines were purchased from the VACSERA and were also a gift from Dr. Mohammed Abu El-Magd, Faculty of Veterinary Medicine, Kafrelsheikh University, Egypt. Lipopolysaccharide (LPS, from *E. coli*) was purchased from Sigma Aldrich, St. Louis, MO, USA. Fetal calf serum (FCS) and penicillin/streptomycin solution were purchased from Hyclone, Logan, UT, USA. RNeasy Mini Kit (#74104) and Quantiscript Reverse Transcription Kit (#205310) were purchased from Qiagen (Hilden, Germany). Finally, 2X Maxima SYBR Green/ROX qPCR Master Mix was obtained from Thermo scientific, Waltham, MA, USA, #K0221. 

### 3.2. In Vitro Cyclooxygenase Inhibition Assay

To assess the inhibitory effects of sulfadiazine on ovine COX-1, human COX-2, and rabbit 5-LOX enzymes, we employed an Enzyme-Immuno-Assay (EIA) kit (Cayman Chemical, Ann Arbor, MI, USA, catalog no. 560131) in accordance with established protocols as previously reported [36,37].

### 3.3. Cell Viability Determination via MTT Assay

HepG2, MCF7, and THLE2 cells were initially seeded at a density of 1 × 10^4^ cells per well (100 μL per well) in DMEM medium. These cultures were then incubated at 37 °C in a 5% CO_2_ environment for 24 h to attain confluency levels ranging from 70% to 80%. Subsequently, different concentrations of sulfadiazine were administered to the wells, resulting in final concentrations spanning from 100 to 3.125 μg/mL. The cells were allowed to culture for an additional 24 h.

After the incubation period, 10 μL of a 12 mM MTT stock solution (5 mg/mL MTT in sterile PBS) was introduced into each well. The plate was incubated for 4 h at 37 °C. Following this, the MTT solution was removed, and the purple formazan crystals that had developed at the well bottoms were dissolved by adding 100 μL of DMSO, allowing for a 20 min incubation. A negative control was included, which involved adding 10 μL of the MTT stock solution to 100 μL of medium alone.

The absorbance at 570 nm was subsequently measured using an ELISA reader (StatFax-2100, Awareness Technology, Inc., Palm City, FL, USA). To determine the proportion of surviving cells, the following formula was applied: (OD of the sulfadiazine-treated sample − OD of the blank)/(OD of the control − OD of the blank) × 100%. Sigmoidal and dose-dependent curves were created to visualize the experimental results. These assays were conducted in triplicate and were repeated in three independent experiments. The concentration of the compounds required to inhibit 50% of the cells (IC_50_) was calculated using the sigmoidal curve.

### 3.4. Stimulation of the Production of Inflammatory Mediators in Cell Lines

HepG2, MCF7, and THLE2 cells were initially seeded in a culture medium comprising DMEM supplemented with 10% FCS and 1% penicillin/streptomycin solution. These cultures were maintained at 37 °C in a 5% CO_2_ environment. The culture medium was refreshed twice a week, and when the cell cultures reached approximately 80% confluence, they were subcultured after trypsinization (0.05%, *w*/*v*). To mitigate mitogenic effects, the cells were pre-cultured in serum-free DMEM for a minimum of 4 h [38].

The cells were exposed to sulfadiazine at a concentration equivalent to 1/10 of the IC_50_ for 1 h. To induce inflammation, 1 μg/mL of LPS was added to the cultures, as previously described [39]. Subsequently, the cells were allowed to incubate for a duration of 24 h. The cells were categorized into three distinct groups, as follows: Group 1 (G1), comprising control cells treated solely with the solvent (DMSO); Group 2 (G2), consisting of cells exposed to 1 µg/mL LPS; and Group 3 (G3), which included LPS-treated cells exposed to 1/10 of the LC50 of sulfadiazine.

### 3.5. Measurement of Inflammatory Markers

Inflammatory marker levels, including TNFα, IL1b, COX-1, COX-2, and 5-LOX, were quantified following established procedures [40]. In short, the cells were seeded at a density of 5 × 10^4^ cells per well in flat-bottomed 96-well plates. Subsequently, sulfadiazine at a concentration equivalent to 1/10 of the LC50 and LPS at a concentration of 1 μg/mL were introduced into the culture medium. The cells were then incubated at 37 °C for a period of 24 h.

After incubation, the culture medium was carefully collected into micro-centrifuge tubes and subjected to centrifugation at 1500 rpm for 10 min. The resulting supernatant was transferred to new micro-centrifuge tubes, and the levels of TNFα, IL1b, COX-1, COX-2, and 5-LOX were determined using an ELISA kit (TNFα, IL1b, COX-1, COX-2, and 5-LOX were determined using enzyme-linked immunoassay (ELISA) kits from Sigma Aldrich (Saint Louis, MO, USA), Abcam (Cambridge, UK), and Aviva Systems Biology (San Diego, CA, USA).

### 3.6. Malondialdehyde Level and Activities of Antioxidant Enzymes

The levels of malondialdehyde (MDA), superoxide dismutase (SOD), catalase (CAT), and glutathione peroxidase (GPX) were quantified using commercial kits, following established protocols [41,42]. To provide a concise overview, when the cultured cells, which had been grown in 25 cm^2^ culture flasks, reached 85% confluence, sulfadiazine at a concentration equivalent to 1/10 of the LC50 was added and allowed to incubate for 1 h. Subsequently, the cells were exposed to 1 µg/mL of LPS for 24 h.

After this incubation period, the cells were rinsed with PBS, harvested from the plates, and suspended in 1 mL of ice-cold PBS (0.1 M, containing 0.05 mM EDTA). The cell suspension was homogenized, and the resulting homogenate was then subjected to centrifugation at 4000× *g* for 10 min at 4 °C. The obtained supernatants were preserved at −80 °C until they were ready for analysis. The protein concentration in each of the supernatant samples was determined using the Bradford assay.

### 3.7. Quantitative Real-Time PCR Analysis

To isolate total RNA from HepG2, MCF7, and THLE2 cells, the RNeasy Mini kit was employed in accordance with the manufacturer’s instructions and established methods [43]. Subsequently, cDNA was synthesized from 4 mg of the total RNA using Quantiscript reverse transcriptase. The resulting cDNA served as a template for evaluating the relative expression of target gene mRNAs, with β-actin serving as an internal reference. To amplify the isolated cDNA, 2X Maxima SYBR Green/ROX qPCR Master Mix was used, following the manufacturer’s protocol, and specific gene primers designed for the purpose. The primer design process was facilitated by the web-based tool, Primer 3 (http://www-genome.wi.mit.edu/cgi-bin/primer/primer3_www.cgi, accessed on 21 March 2020), which utilized published human sequences as a basis.

The reaction volume and qPCR thermal conditions were consistent with those previously described [44]. Upon completion of the final cycle, the temperature was increased from 60 to 95 °C to generate a melt curve. The relative change in gene expression was presented as a fold change, determined using quantities at the critical threshold (Ct) and the 2^−∆∆Ct^ method [45] (Table 5).

### 3.8. Statistical Analysis

The data were presented as mean ± standard error (S.E.), and the assessment of statistical significance was conducted through one-way analysis of variance (ANOVA) utilizing SPSS software version 18.0 from the year 2011. Individual comparisons were subsequently made using Duncan’s multiple range test (DMRT). Statistical significance was determined when the *p*-value was lower than 0.05.

### 3.9. Molecular Modeling

#### 3.9.1. Computational Tools

Computational simulations were performed utilizing the Maestro graphical user interface developed by Schrödinger, LLC, based in New York (Maestro, version 11.8. (2018) Schrödinger, LLC, New York, NY, USA) [46]. Molecular dynamic simulation studies were conducted on a desktop workstation running the Linux Ubuntu operating system, with the added support of an RTX 5000 graphics card.

#### 3.9.2. Crystal Structures

The crystal structures of the enzymes under investigation, namely COX-1, COX-2, and 5-LOX, were obtained from the Research Collaboratory for Structural Bioinformatics (RCSB) Protein Data Bank (PDB) and corresponded to the following PDB IDs: 5WBE, 3LN1, and 3V99, respectively [47,48].

#### 3.9.3. Protein Preparation

All of the crystal structures were meticulously preprocessed with the assistance of the Schrödinger Maestro’s Protein Preparation Wizard tool. This preparation and minimization process was executed under a pH of 7.4, rectifying ionization states as necessary. Polar hydrogens were incorporated, and non-essential water molecules were excluded from the structures. The entire protein structure was subjected to a thorough minimization and optimization procedure utilizing the OPLS3 force field. This optimization aimed to enhance protein energies and eliminate any steric hindrance, with a default root mean square deviation (RMSD) value of 0.30 Å applied to non-hydrogen atoms [49].

#### 3.9.4. Ligand Library Preparation

For ligand preparation, Maestro Ligprep was utilized. The structures initially obtained in SDF format were converted to the 3D Maestro format. Optimal chirality and ionization states were established at a physiological pH of 7.4 ± 2.0 by employing Epik [50]. Throughout this procedure, a series of refinements and adjustments were applied to the ligand structures. Eventually, the geometries were fine-tuned using the OPLS3 force field. These refined conformations were then used as the initial input structures for the docking simulations.

#### 3.9.5. Binding Pocket Determination 

In Schrödinger’s Maestro, the binding pocket was pinpointed based on the co-crystallized ligand within the workspace. These identified and selected binding pockets were employed to construct a docking grid using Maestro’s Glide module for the subsequent docking studies. The receptor grids were created utilizing the prepared protein structures, and these grids were centered on the specific binding pockets identified for each protein. The generation of a receptor grid involved employing a van der Waals (vdw) radius scaling factor of 1.00 and a partial charge cut-off of 0.25. The binding sites were encompassed within a grid box of 20 Å^3^ with default parameters and no constraints.

#### 3.9.6. Validation of Molecular Docking

The validation of the molecular docking process was carried out by assessing Maestro Glide’s capability to predict conformations that closely resemble the experimental conformations, as previously documented in the literature [51,52,53]. The crystallographic ligands were subjected to docking within their respective targets, utilizing the identical docking protocol applied to the potential ligands. Among the docked poses, the one displaying the most negative binding energy for each target was identified. This selected pose was then structurally aligned with the conformation depicted in the crystallographic structure using Maestro’s structure superimposition feature, and the root mean square deviation (RMSD) of the alignments was computed.

#### 3.9.7. Molecular Docking

The ligands underwent docking procedures employing the extra precision mode (XP) without any constraints, and specific parameters included a van der Waals (vdw) radius scaling factor of 0.80 and a partial charge cut-off of 0.15. To estimate binding affinity and rank ligands, GlideScore, implemented in the Glide software (Maestro, version 11.8. (2018) Schrödinger, LLC, New York), was employed. The XP Pose Rank was used to identify and select the best-docked pose for each ligand. Subsequently, the final list of compounds was subjected to in-depth analysis by considering binding scores and conducting a comprehensive examination of all binding interactions.

#### 3.9.8. Molecular Dynamics Simulation Studies

The molecular dynamics (MD) simulation was executed utilizing the Desmond Module within Schrödinger’s Maestro platform. Initially, the docked complex underwent minimization through the protein preparation wizard, and the resulting minimized complex was then prepared for MD simulation using the system builder application within Desmond. The MD simulation environment featured a solvent system based on water using the TIP3P water model.

A simulation box with an orthorhombic shape and a buffer parameter extending 10 angstroms from the protein surface was generated. The entire system was neutralized by calculating and introducing the requisite number of counter ions. Additionally, 0.15 M NaCl was included to achieve an isosmotic condition. The MD simulation was conducted under conditions of 300 K for temperature and 1.013 bar for atmospheric pressure. The simulation was run for a total duration of 100 nanoseconds, and 1000 frames were saved to compile the trajectory data. The analysis of the simulation and the presentation of the results were carried out using the simulation interaction diagram tool provided by Desmond.

## 4. Conclusions

Our study aimed to evaluate the activity profile of sulfadiazine and assess its repurposing suitability as a potential anticancer drug. In vitro data regarding the impact of sulfadiazine treatment on the expression of pro-inflammatory mediators and its role in mitigating oxidative stress suggest that sulfadiazine operates through various mechanisms to suppress inflammation and oxidative stress. Notably, we report on the inhibitory effects of sulfadiazine on the cellular proliferation of breast and liver cancer cells, positioning it as a promising therapeutic agent for both cancers. Our findings contribute to the growing body of literature aimed at validating the COX/LOX pathway as a potential target in the quest for innovative cancer therapeutics.

Molecular modeling studies corroborate our biological results and propose that sulfadiazine holds promise as a COX inhibitor with favorable COX-2 selectivity and a more balanced impact on the COX/LOX pathway, rendering it a potential candidate for repurposing as a prospective anticancer drug. Furthermore, our results underscore the highly promising COX/LOX inhibition pattern of sulfadiazine, warranting further investigation to validate this target’s therapeutic potential in preclinical and clinical contexts. Future research will also delve into confirming the antiproliferative activity of sulfadiazine in clinical studies. 

## Figures and Tables

**Table 1:** In vitro Cox-1, Cox-2 and 5-Lox inhibitory activity of sulfadiazine and celecoxib.

Compound	COX-1 IC_50_ (μM) *	COX-2 IC_50_ (μM) *	5LOX IC_50_ (μM) *
Sulfadiazine	18.4	5.27	19.1
Celecoxib	5.9	1.94	16.7

* The concentration of test compound that produces 50% inhibition of COX-1, COX-2, 5-LOX enzyme; the result is the mean of three values obtained via assay of enzyme kits.

**Table 2:** Inhibition IC_50_ of sulfadiazine and cisplatin on HEPG2, MCF7, and THLE2 cell lines.

Compound	HEPG2 IC_50_ (μM)	MCF7 IC_50_ (μM)	THLE2 IC_50_ (μM)
Sulfadiazine	245.69 ± 4.1	215.68 ± 3.8	4159 ± 90.5
Cisplatin	66.92 ± 1.8	46.83 ± 1.3	2144 ± 95.3

**Table 3:** Glide scores and selectivity indices for the screened antibacterial sulfonamides against COX-1, COX-2, and 5-LOX.

	Glide Score			Selectivity COX-1/COX-2/5-LOX
	COX-1	COX-2	5-LOX	
Sulfadiazine	−4.9	−6.1	−5.3	1:1.3:1.1
Sulfamerazine	−6.1	−5.7	−5.3	1.6:1.1:1
Sulfathiazole	−5.6	−5.3	−5.2	1.1:1:1
Sulfameter	−5.4	−5.7	−5.1	1.1:1.1:1
Sulfadimidine	−5.2	−5.5	−5.1	1:1.1:1
Sulfadoxine	−5.3	−5.3	−4.9	1.1:1.1:1
Sulfaguanidine	−4.8	−5.9	−4.8	1:1.2:1
Sulfabenzamide	−5.6	−7.3	−4.4	1.2:1.7:1
Celecoxib	−10.1	−11.6	−5.3	1.9:2.2:1

**Table 4:** Experimental IC_50_ and calculated binding affinities of sulfadiazine and celecoxib with COX-1, COX-2, and 5-LOX.

	Experimental IC_50_ (μM) *				Calculated Binding Affinity			
	COX-1	COX-2	5-LOX	Selectivity COX-1/COX-2/5-LOX	COX-1	COX-2	5-LOX	Selectivity COX-1/COX-2/5-LOX
Sulfadiazine	18.4	5.27	19.1	1:3.6:1	−4.9	−6.1	−5.3	1:1.3:1.1
Celecoxib	5.9	1.94	16.7	3:8.6:1	−10.1	−11.6	−5.3	1.9:2.2:1

* The concentration of test compound that produces 50% inhibition of COX-1, COX-2, 5-LOX enzyme; the result is the mean of three values obtained via assay of enzyme kits.

**Table 5:** Forward and reverse primers used in qPCR.

Gene	Forward Primer (^/^5 ------ ^/^3)	Reverse Primer (^/^5 ------ ^/^3)
TNFa	CCCAGGGACCTCTCTCTAATC	ATGGGCTACAGGCTTGTCACT
IL1β	ACAGATGAAGTGCTCCTTCCA	GTCGGAGATTCGTAGCTGGAT
Cox2	CCCTTGGGTGTCAAAGGTAA	GCCCTCGCTTATGATCTGTC
COX-1	AAGGAGATGGCAGCAGAGTT	GTGGCCGTCTTGACAATGTT
5LOX	ATTGCCATCCAGCTCAACCAAACC	TGGCGATACCAAACACCTCAGACA
β-actin	CGACATCAGGAAGGACCTGTATGCC	GAAGATTCGTCGTGAAAGTCG

## Data Availability

Data is contained within the article and Supplementary Materials.

## Supplementary Materials

The following supporting information can be downloaded at: https://www.mdpi.com/article/10.3390/ph17020189/s1, Figure S1: Illustration of the linear amplification curves representing the Ct values of inflammation-related genes (TNFa, IL1b, COX-1, COX-2, 5LOX) in LPS-inflamed HepG2, MCF7, and THLE2 cells. The housekeeping gene encoding β-actin was employed as an internal reference.
