# Supplementary material for: Sulfadiazine Exerts Potential Anticancer Effect in HepG2 and MCF7 Cells by Inhibiting TNFα, IL1b, COX-1, COX-2, 5-LOX Gene Expression: Evidence from In Vitro and Computational Studies
Source: Pharmaceuticals (Basel). 2024 Jan 31;17(2):189. doi: 10.3390/ph17020189 (PMC10891904; doi:10.3390/ph17020189)
Supplement: Supplementary file 1 [file pharmaceuticals-17-00189-s001.zip › pharmaceuticals-2820426-supplementary.pdf]

	HepG2	MCF7	THLE2
**TNFα**	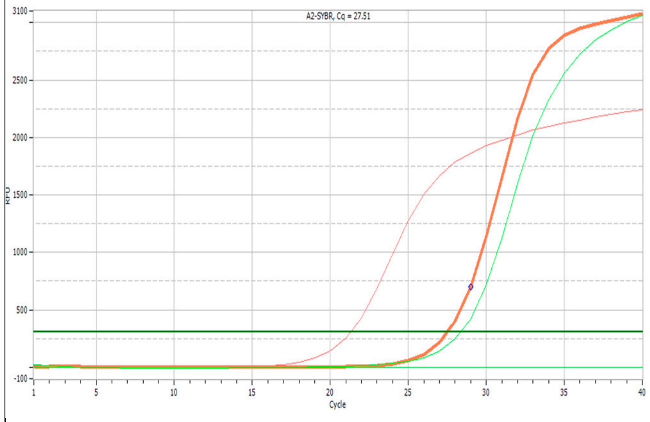	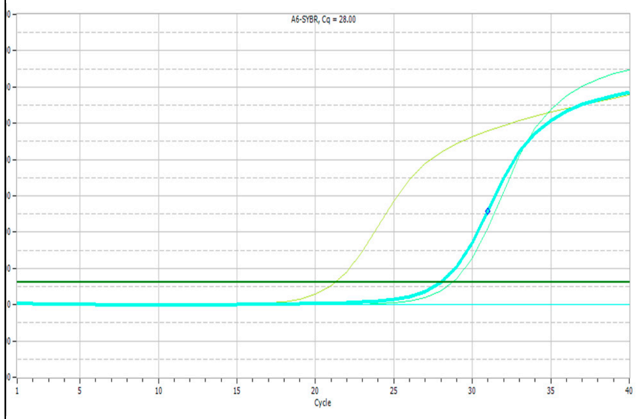	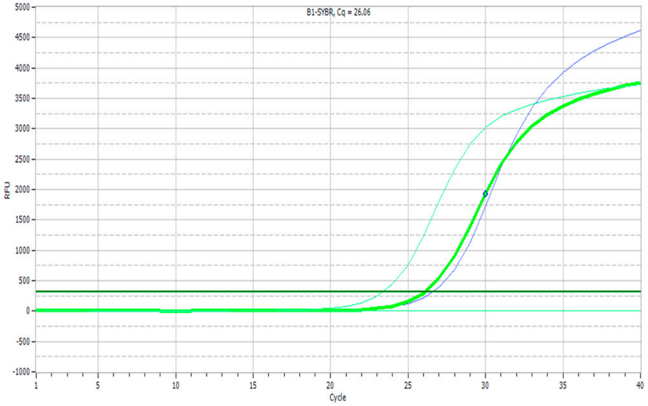
**IL1β**	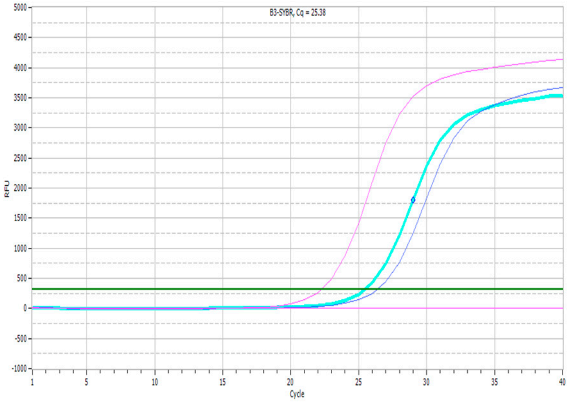	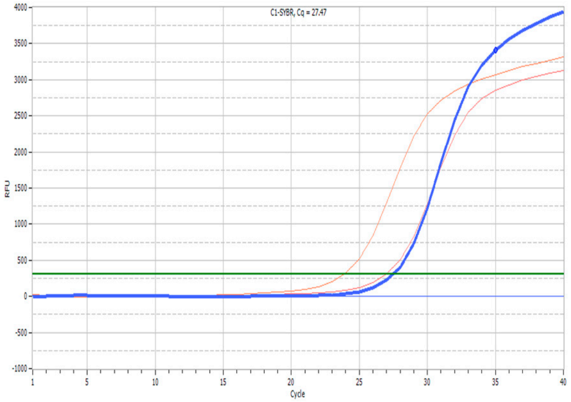	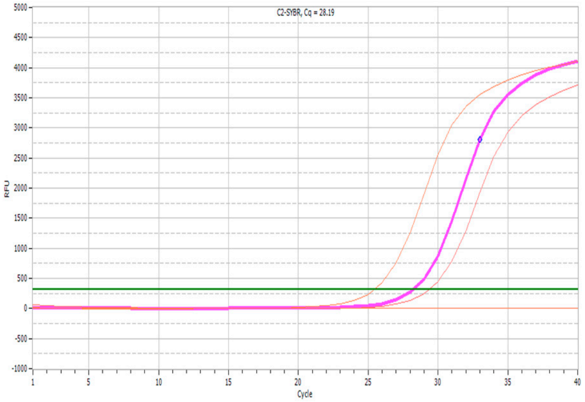

COX-1	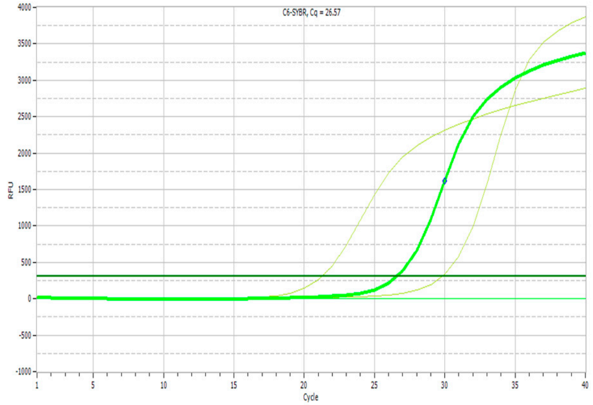C6-SYBR, Cq = 26.57<br>RFU<br>Cycle	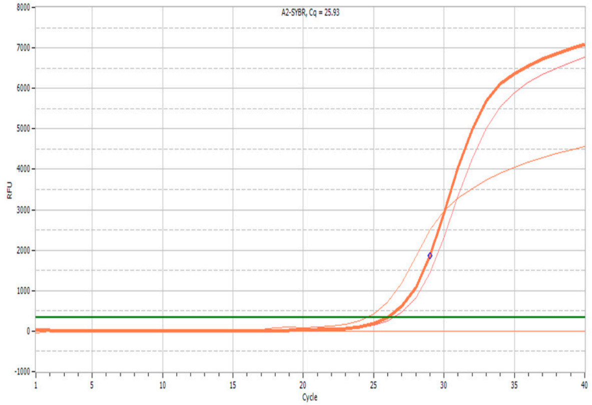A2-SYBR, Cq = 25.93<br>RFU<br>Cycle	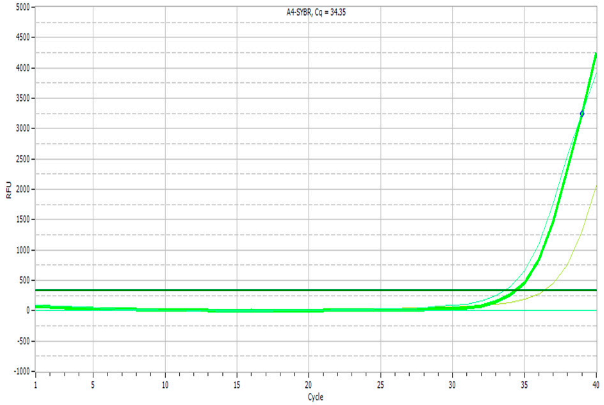A4-SYBR, Cq = 34.35<br>RFU<br>Cycle
COX-2	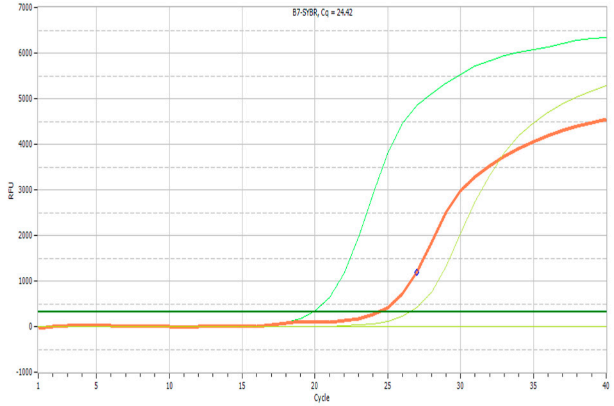B7-SYBR, Cq = 24.42<br>RFU<br>Cycle	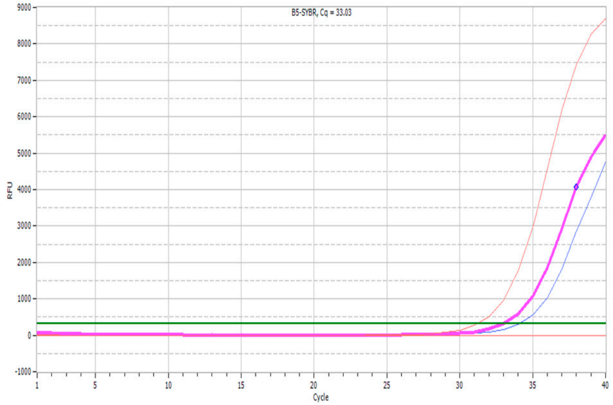B5-SYBR, Cq = 33.03<br>RFU<br>Cycle	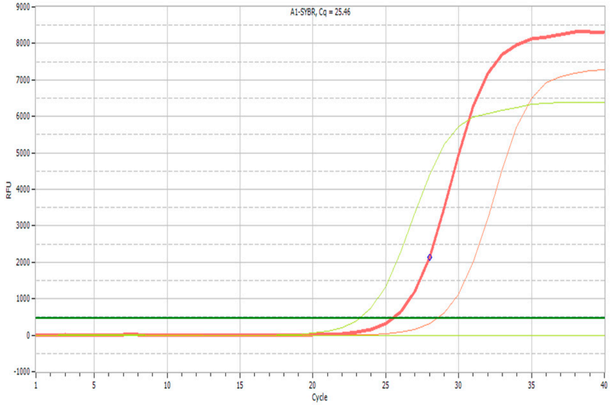A1-SYBR, Cq = 25.46<br>RFU<br>Cycle

5LOX

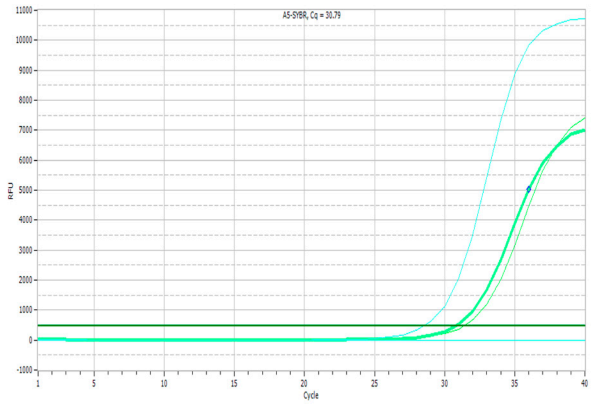

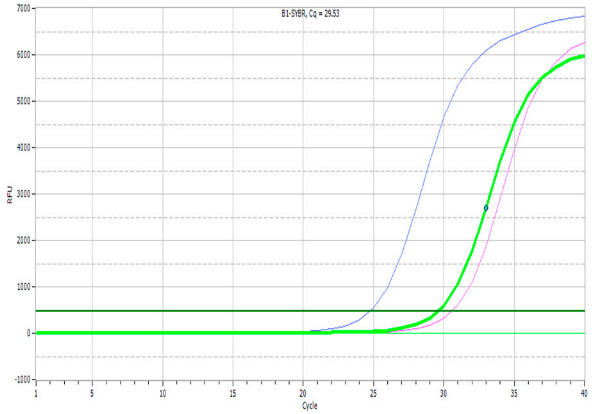

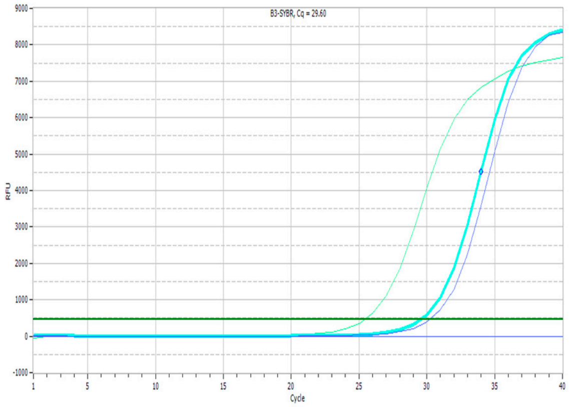

Figure S1. The set of curves obtained from G1 (control, vehicle-treated cells), G2 (untreated LPS-inflamed cells), and G3 (sulfadiazine-treated LPS-inflamed cells) for each of the five inflammation-related genes (TNFα, IL1β, COX-1, COX-2, 5LOX), plotted against the cycle number (x-axis) and fluorescence intensity (y-axis). The data was obtained from Real time PCR (qPCR) analysis.
